# A decision-driven framework for the mass spectrometry analysis of previously uncharacterized protein modifications

**DOI:** 10.1016/j.xpro.2026.104567

**Published:** 2026-05-28

**Authors:** Yiying Zhu

**Affiliations:** 1 Department of Chemistry, Tsinghua University, Beijing 100084, China

**Keywords:** Proteomics, Mass Spectrometry, Chemistry

## Abstract

Identification of unknown protein modifications remains challenging when the modification chemistry or site is not defined in advance. Conventional workflows often rely on predefined modification lists or enrichment strategies that assume prior knowledge of modification type and may therefore bias discovery toward annotated post-translational modifications (PTMs). This primer outlines a decision-driven analytical framework for investigating previously uncharacterized modifications using bottom-up (liquid chromatography-tandem mass spectrometry) LC-MS/MS that emphasizes chemistry-informed hypothesis generation, iterative refinement of candidate modification search space, integration of experimental controls, and targeted data interpretation. Rather than presenting a single prescriptive workflow, the guide highlights key decision points in experimental design, acquisition strategy, and database search configuration that influence confident identification and residue-level localization. The framework is broadly applicable to drug-induced covalent adducts, chemically introduced modifications, as well as endogenous modifications arising across diverse experimental and biological contexts.

## Introduction

Protein function is regulated not only by primary amino acid sequence but also by a wide spectrum of chemical modifications that occur after translation or through interactions with endogenous metabolites, reactive species, or exogenous compounds. Post-translational modifications (PTMs) influence protein activity, stability, localization, and molecular interactions, thereby expanding functional proteome diversity far beyond genomic encoding.^1^^,^^2^^,^^3^^,^^4^ Large-scale proteomics studies have revealed extensive PTM landscapes across organisms, highlighting dynamic regulatory networks that respond to developmental, physiological, environmental, and chemical perturbations and demonstrating that many modification events remain context-dependent, low in stoichiometry, chemically heterogeneous, or incompletely annotated.^5^^,^^6^

Mass spectrometry (MS) has become the dominant analytical platform for identifying protein modifications due to its sensitivity, molecular specificity, and ability to localize modification sites at the amino acid level. Bottom-up proteomics, in which proteins are enzymatically digested prior to LC–MS/MS analysis, is widely used to map modification sites in complex biological samples.^7^^,^^8^ Advances in high-resolution mass analyzers, chromatographic separation, tandem MS fragmentation methods, and quantitative proteomics have enabled proteome-wide characterization of canonical PTMs including phosphorylation, acetylation, ubiquitination, methylation, and glycosylation.^6^^,^^9^^,^^10^^,^^11^^,^^12^^,^^13^^,^^14^ However, workflows optimized for large-scale, annotation-driven PTM mapping do not directly address analytical scenarios in which modification identity and site are not specified *a priori* for a defined protein target. In such cases, search-space design, acquisition strategy, and interpretation criteria require deliberate constraint and iterative refinement rather than broad proteome-wide discovery.

Identification of unknown protein modifications remains a significant analytical challenge. Conventional database search strategies rely on predefined modification lists and therefore exhibit inherent bias toward annotated PTMs.^15^ Although open and mass-tolerant search approaches expand the detectable mass space,^16^^,^^17^^,^^18^^,^^19^^,^^20^ they typically require large datasets and often produce extensive candidate mass shifts that necessitate iterative chemical interpretation to achieve confident identification. In parallel, proteome-wide discovery strategies increasingly combine open-search approaches with large-scale quantitative proteomics workflows to survey modification events across complex biological samples. However, when the objective is to localize modification sites on defined protein targets, targeted analytical strategies that constrain the search space and incorporate chemical reasoning remain essential for confident interpretation.

In many experimental contexts, protein modifications arise from chemical exposures, reactive metabolites, environmental factors, and unintended reactions during sample handling. Such modifications may occur at low stoichiometry, exhibit heterogeneous chemical outcomes, or display instability during sample preparation and MS analysis, complicating confident identification. Challenges are particularly pronounced in chemical biology and drug discovery applications, where covalent inhibitors, electrophilic probes, and reactive metabolites can generate heterogeneous adducts with diverse chemical outcomes and variable stability.^21^^,^^22^^,^^23^^,^^24^ Activity-based protein profiling and bioorthogonal chemistries have enabled systematic interrogation of reactive amino acid residues, yet confident site localization frequently depends on tailored analytical workflows and enrichment strategies.^25^^,^^26^^,^^27^^,^^28^^,^^29^ In addition, the physicochemical diversity of modifications—including differences in mass, charge, hydrophobicity, fragmentation behavior, and stability—directly influences detectability and localization, making the selection of appropriate acquisition and analysis strategies a critical decision point.^30^

A central theme emerging from these challenges is that analysis of protein modifications whose identity or site is not known in advance is inherently decision-driven rather than procedural. Experimental design, sample preparation, acquisition strategy, and database search constraints must be adapted iteratively based on evolving evidence, available prior knowledge, and specific research objectives. Decisions regarding control design, enrichment strategies, fragmentation methods, and search parameters can strongly influence the ability to detect and localize modification sites. Confident interpretation typically requires integration of computational identification with manual spectrum evaluation, comparison to matched controls, and orthogonal validation strategies such as synthetic peptide standards.^31^

**Figure 1:**
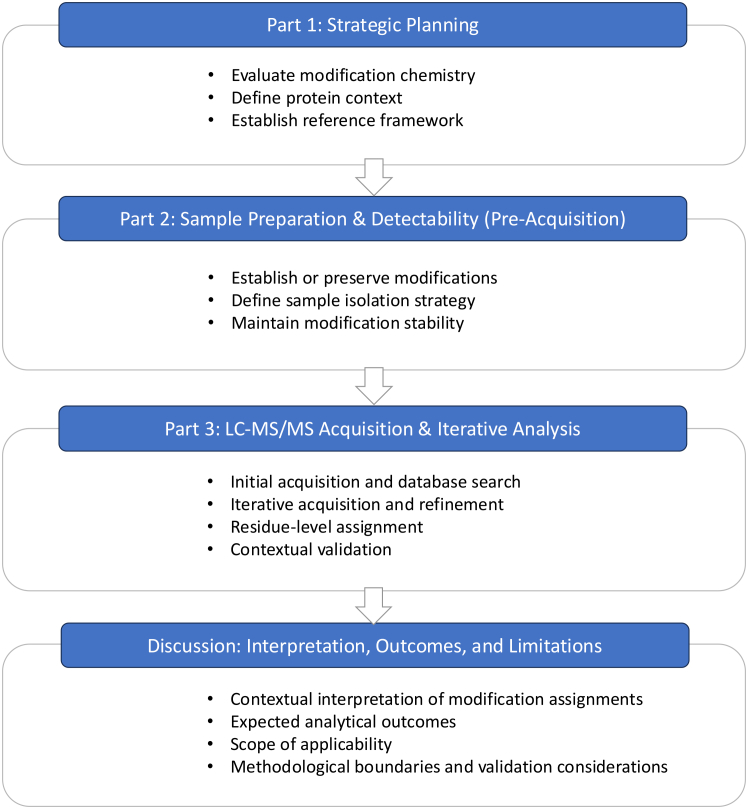
Decision framework for targeted analysis of previously uncharacterized protein modifications Schematic overview of a decision-driven workflow for the analysis of previously uncharacterized protein modifications. The framework is organized into three interconnected stages: strategic planning, sample preparation and detectability (pre-acquisition), and LC–MS/MS acquisition with iterative data analysis, followed by contextual interpretation. The process begins with defining modification chemistry, protein context, and an appropriate reference framework, which guide subsequent experimental design. Pre-acquisition considerations focus on preserving modification stability and ensuring detectability through appropriate sample isolation strategies. LC–MS/MS analysis is performed in an iterative manner, combining data acquisition, database searching, and refinement to support residue-level assignment. Final interpretation integrates analytical evidence with experimental context to evaluate the plausibility and significance of modification assignments, while explicitly considering methodological limitations and scope of applicability.

Given these challenges, there is a need for conceptual guidance that emphasizes decision-making rather than prescriptive workflows. This Primer discusses an approach for investigating protein modifications whose identity or site is initially unknown, using bottom-up LC–MS/MS. The framework emphasizes chemistry-informed hypothesis generation, iterative refinement of candidate modification space, integration of experimental controls, and targeted data interpretation (Figure 1). By highlighting key considerations across experimental design, acquisition strategy, and data analysis, this guide aims to support researchers in developing tailored strategies for identifying and localizing previously uncharacterized modifications. The workflow described here naturally spans interdisciplinary environments: Strategic planning, laboratory implementation, and analytical refinement within mass spectrometry facilities.

## Part 1: Strategic planning

The primary objective of this framework is the confident identification and residue-level localization of previously uncharacterized protein modifications. Experimental planning should therefore prioritize detectability, interpretability, and comparative validation rather than early quantification or mechanistic extrapolation. Because analysis of unknown protein modifications is discovery-oriented, decisions made at the outset strongly influence whether modifications remain detectable and interpretable during downstream LC–MS/MS analysis. Routine instrument performance verification and basic sample-level controls are assumed as standard practice and are therefore not discussed in detail here. Instead, the framework focuses specifically on modification-dependent analytical decisions rather than general mass spectrometric troubleshooting.

### Evaluate modification chemistry

A logical starting point is the assessment of the suspected modification itself. Consideration of plausible reaction chemistry—including reactive functional groups, potential transformation pathways, anticipated stability, and candidate residues— determines analytical tractability and defines the boundaries of feasible detection strategies. These chemical properties guide downstream hypothesis construction and search-space design. If a modification is chemically labile, heterogeneous, or prone to transformation under standard processing conditions, preservation or stabilization strategies should be incorporated during planning. Together with the defined protein context and reference framework described below, this chemical assessment establishes the analytical boundaries within which modification identification becomes feasible.

### Define the protein context

Once modification properties are evaluated, the experimental context of the protein must be defined. This decision should be made during strategic planning. Purified or recombinant protein systems enable controlled reaction environments and simplified background, whereas investigation of proteins within complex biological matrices preserves physiological relevance but introduces endogenous modifications and increased analytical complexity. Preliminary orthogonal assessment of modification presence may help confirm biological inducibility prior to peptide-level mass spectrometry analysis. Compatibility with downstream LC–MS/MS should be anticipated at this stage whenever possible. Importantly, defining the protein context at this stage clarifies whether enrichment or fractionation will be performed upstream or implemented within the analytical workflow, and informs later requirements for digestion strategy, cleanup, and achievable sequence coverage. This contextual decision ultimately informs later requirements for enrichment, digestion strategy, and analytical depth.

### Establish the reference framework

Interpretation of candidate modifications depends on appropriate reference conditions, which vary according to experimental purpose. In biologically driven studies, comparison between perturbation and baseline states may clarify condition-dependent modifications. In chemically driven workflows, negative controls such as omission of reactive reagent, use of inactive analogs, or competition conditions may be more relevant. The choice of reference framework should align with the intended question—whether assessing biological modulation, mapping covalent interaction sites, or identifying chemically induced adducts. A clear definition of this comparison architecture is essential for robust and interpretable modification assignment.

### Integration across technical domains

Because assessment of reaction chemistry and analytical feasibility spans distinct technical domains, integration of chemical, biological, and mass spectrometry specialists is critical.^4^^,^^21^^,^^23^^,^^25^^,^^28^ Conceptual planning should be established prior to experimental execution to ensure alignment between modification chemistry, sample preparation, and analytical strategy. Depending on the experimental setting, modification generation, orthogonal validation, and LC–MS/MS analysis may be performed within a single laboratory or distributed across different technical environments. In core facility settings, these stages are often implemented collaboratively across specialized teams. Regardless of implementation, maintaining alignment across these stages is critical for successful modification identification.

Together, evaluation of modification properties, definition of protein context, and establishment of a clear comparison framework form the conceptual foundation for downstream sample processing and analytical interpretation.

Strategic planning defines the analytical trajectory of the workflow, including expected modification chemistry, protein context, and comparison framework. In chemically defined systems, such as drug-directed covalent modifications, planning is often guided by known or hypothesized reaction mechanisms, enabling prediction of modification mass, target residues, and potential analytical readouts. These systems may be implemented in purified proteins or cellular contexts, but the defining feature is that modification formation is controlled and chemically interpretable.

In contrast, endogenous or condition-dependent modification scenarios rely more heavily on comparative experimental design. In such cases, modification presence is typically inferred through comparison between matched conditions (e.g., stimulated versus unstimulated samples), and the underlying chemistry may be less well defined or only partially understood. As a result, modification detection depends on differential evidence rather than direct prediction or predefined readouts.

**Figure 2:**
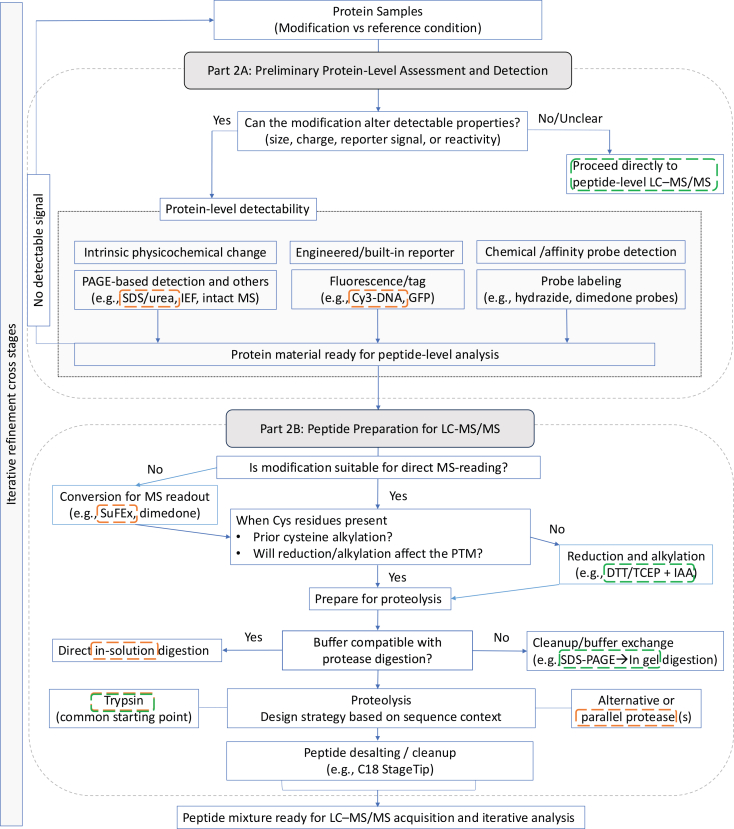
Decision framework for detectability assessment and sample preparation in analysis prior to LC-MS/MS analysis Schematic overview of decision-making strategies for sample preparation and detectability assessment in the analysis of previously uncharacterized protein modifications. The workflow is divided into two interconnected components: preliminary protein-level assessment (Part 2A) and peptide preparation for LC–MS/MS analysis (Part 2B). The process begins with evaluation of whether the modification induces detectable physicochemical changes at the protein level (e.g., size, charge, or probe reactivity), guiding the use of orthogonal detection methods such as gel-based approaches, intact mass analysis, or affinity/chemical probe labeling. When direct detectability is limited, alternative strategies—including probe-based conversion or engineered reporters—may be applied to enable downstream analysis. Sample preparation proceeds through proteolysis and peptide cleanup, with considerations for buffer compatibility, protease selection, and potential effects of reduction and alkylation on modification stability. The framework emphasizes flexibility in adapting preparation strategies based on modification properties, including the use of alternative or parallel proteases when needed. Dashed overlays highlight representative applications of the framework. The SuFEx-based example (orange) illustrates a chemically defined covalent modification system in which decision-making is concentrated in protein-level detectability and sample preparation, including SDS–PAGE assessment and conversion of bulky adducts into MS-compatible forms. In contrast, the endogenous modification example (green), represented by a citrullination-like scenario, shows a workflow in which protein-level detectability is limited and analysis proceeds directly from purified protein to peptide-level processing (e.g., in-gel digestion), bypassing modification-specific detection at the protein level. Iterative refinement may occur throughout the workflow and connects to downstream LC–MS/MS acquisition and interpretation (Figure 3).

These distinct planning strategies shape downstream decisions in detectability assessment, sample preparation, and LC–MS/MS analysis, as illustrated in Figure 2.

## Part 2: Preliminary protein-level assessment and sample preparation for LC-MS/MS analysis

### Rationale

LC–MS/MS analysis of unknown modifications is iterative, analytically demanding, and resource-intensive. Acquisition parameters, database search space, and interpretation criteria often require coordinated refinement before confident site localization is achieved. When feasible, rapid protein-level assessment can confirm whether modification-dependent changes have occurred and clarify expectations regarding abundance and stability. Such assessment functions as an efficiency filter, distinguishing failed modification generation from downstream analytical limitations.

Following this evaluation, protein material must be processed in a manner that preserves modification integrity while ensuring compatibility with peptide-level LC–MS/MS analysis. Together, these steps bridge experimental planning and mass spectrometric interrogation.

As illustrated in Figure 2, these decisions can follow distinct paths depending on modification context, with representative examples highlighting differences between chemically defined and endogenous modification scenarios.

## Part 2A: Preliminary protein-level assessment

Preliminary assessment provides an efficiency filter before committing to iterative LC–MS/MS analysis. When modification-dependent physicochemical changes are expected to be detectable, rapid protein-level methods can confirm modification generation and inform expectations regarding abundance and stability.

### Protein preparation

Preparation strategy should reflect the protein context defined during strategic planning and is typically established prior to analytical workflow execution. Affinity-purified or recombinant proteins can often be processed directly with minimal background interference. In contrast, proteins analyzed within complex cellular or tissue matrices frequently require enrichment, selective isolation, or, in rare cases, fractionation,^32^ to reduce analytical complexity, consistent with the comparison framework established in Part 1.

Samples representing predefined comparison conditions should be processed in parallel through all subsequent steps, including enrichment, conversion (if applied), digestion, and peptide cleanup. Consistent handling order, timing, and reagent exposure ensure that observed differences reflect the intended analytical variable rather than technical variability.

### Evaluating protein-level detectability

Assessment begins by considering the predicted consequences of the modification, including potential effects on molecular weight, net charge, binding properties, chemical reactivity, and expected stoichiometry relative to assay sensitivity. These features determine whether the modification can be detected at the intact-protein level prior to peptide-level LC–MS/MS analysis.

#### Intrinsic physicochemical property-based detection

Some modifications alter fundamental physicochemical properties of proteins, including apparent molecular weight or net charge. When such changes occur at appreciable abundance, electrophoretic or intact-mass measurements can provide rapid preliminary evidence that a modification event has occurred.

Gel electrophoresis can reveal mobility shifts, additional bands, or altered band patterns when modifications introduce substantial mass differences or covalent conjugates. Most commonly, SDS–PAGE is used to assess changes in apparent molecular weight resulting from large adducts or protein conjugates. In systems involving protein–nucleic acid conjugates, denaturing urea–PAGE can resolve conjugated species from unmodified components based on altered electrophoretic mobility.

Charge-altering modifications can also be detected using isoelectric focusing (IEF), which separates proteins according to their isoelectric point (pI). Differences in glycosylation, particularly variation in terminal sialylation, can shift protein charge and generate distinct pI distributions. Glycoform heterogeneity of prostate-specific antigen (PSA), for example, reflects variation in glycan composition that influences electrophoretic behavior.^33^^,^^34^

When purified or enriched protein is available, intact mass measurement using LC–MS, MALDI–TOF, or direct infusion MS can provide more direct evidence of modification by revealing reproducible mass differences between modified and unmodified species and suggesting modification multiplicity.^4^^,^^35^ Compared with electrophoretic methods, intact MS provides higher specificity because the observed mass shift directly reflects the molecular change rather than altered migration behavior.

Although these approaches do not provide residue-level identification, they offer rapid orthogonal evidence that modification events are present and help guide subsequent peptide-level LC–MS/MS analysis.

#### Engineered reporter signal detection

In some experimental systems, the modification or conjugate carries a built-in reporter signal that enables direct visualization without additional derivatization. Such signals may include fluorescent labels, chromophores, or genetically encoded reporters introduced during chemical or molecular engineering steps.

For example, fluorescent protein reporters such as GFP have long been used to visualize protein localization and interactions directly in cells.^36^ Similarly, in DNA-encoded ligand or covalent screening platforms, oligonucleotide tags bearing fluorophores can enable direct visualization of DNA–protein conjugates following electrophoresis, facilitating rapid isolation of modified species prior to downstream characterization. In one such approach, Cy3-labeled DNA libraries allowed direct visualization of DNA–protein conjugates on denaturing gels, enabling gel extraction of modified complexes before sequencing or LC–MS analysis.^37^^,^^38^

Although these signals typically arise from engineered reporters rather than endogenous protein chemistry, they can provide rapid confirmation that a modification or conjugation event has occurred prior to peptide-level analysis.

#### Chemical reactivity or affinity-based detection

When a modification introduces or exposes a distinct chemical functionality, selective probes or affinity reagents can enable sensitive detection even when the modification is present at low stoichiometry. In some cases, antibodies recognizing specific modification classes or chemical motifs are available and can be used to assess whether a modification occurs at the protein level, even when the precise modification site remains unknown. However, such reagents are not available for many modification types. In these situations, chemical probes that react selectively with defined functional groups provide an alternative strategy for detecting modified proteins.

For example, protein carbonylation generates aldehyde or ketone groups that can be derivatized using hydrazide-based probes such as biotin–hydrazide, enabling affinity-based visualization or capture of oxidatively modified proteins prior to LC–MS/MS analysis.^39^^,^^40^ Similarly, cysteine sulfenic acid intermediates can be selectively trapped using dimedone-derived probes, allowing stabilization and detection of transient oxidative modifications.^41^ In many probe-based workflows, the reacting probe also introduces a reporter handle—such as a fluorescent tag or biotin—allowing visualization or affinity-based detection of labeled proteins before subsequent peptide-level LC–MS/MS analysis.

More broadly, chemoproteomic strategies often employ activity-based probes that react with specific classes of reactive residues or enzyme active sites. In activity-based protein profiling (ABPP), reactive probes containing electrophilic warheads and reporter handles enable covalent labeling of proteins in complex proteomes, followed by fluorescence-based or affinity-based visualization of labeled species.^25^ Subsequent LC–MS/MS analysis can then identify the labeled proteins and localize the modified residues. Related strategies use bioorthogonal reporter probes containing alkyne or azide handles to tag proteins modified by reactive electrophiles or other chemical perturbations. These handles can subsequently be coupled to reporter tags through click chemistry, enabling visualization and LC–MS/MS identification of modification sites.^28^^,^^42^^,^^43^ More generally, chemical derivatization may generate simplified residue-level mass signatures that facilitate downstream LC–MS/MS identification. Because such approaches primarily support peptide-level interpretation rather than initial protein-level assessment, they are only briefly noted here and are discussed in the subsequent LC–MS/MS analysis section.

### Interpreting negative or ambiguous results

Absence of a detectable mobility shift, intact mass change, or affinity signal does not exclude modification. Interpretation should reflect whether the anticipated physicochemical consequence was expected to be detectable under the selected assay conditions.

If a substantial shift or reporter signal was strongly predicted but not observed, reaction conditions, or modification generation should be reassessed, including modification efficiency, stability, or detection sensitivity. Conversely, when the modification is expected to be subtle, heterogeneous, low in stoichiometry, or labile, intact-level methods may lack sufficient sensitivity, and direct peptide-level LC–MS/MS analysis may be more informative.

Interpretation should be made relative to a defined reference condition appropriate to the experimental question (e.g., omission of reactive reagent, inactive analog, or untreated sample), ensuring that observed signals reflect modification-dependent changes rather than background variation.

## Part 2B: Peptide preparation for LC-MS/MS analysis

Following assessment (or when assessment is not feasible), protein material must be processed into peptide mixtures that preserve modification information and are compatible with chromatographic separation and mass spectrometric analysis.

### Conversion for MS readout (when necessary)

When intact modifications are chemically unstable, bulky, heterogeneous, or poorly fragmenting, chemical conversion to a simplified residue-level mass signature can improve detectability and facilitate confident site localization. Such strategies reduce structural complexity while preserving positional information and enabling focused database searching under defined mass constraints.

In some cases, unstable modification intermediates can be chemically trapped prior to analysis. For example, cysteine sulfenic acid modifications are often stabilized through reaction with dimedone-derived reagents, converting transient oxidative intermediates into stable adducts that can be detected and localized by LC–MS/MS. In other situations, heterogeneous or structurally complex adducts can be converted into simplified residue-level markers prior to peptide analysis. Sulfur(VI) fluoride exchange (SuFEx)–based strategies provide one illustration,^38^ in which reaction-derived conjugates are converted into simplified residue signatures, facilitating peptide-level identification by LC-MS/MS. More broadly, similar conversion logic applies whenever removal or transformation of unstable or heterogeneous moieties improves peptide fragmentation behavior and analytical interpretability.

Because conversion alters the original adduct structure, the resulting signal represents a chemically derived marker rather than the intact modification itself. Accordingly, conversion strategies should be guided by plausible reaction chemistry and interpreted within the defined comparison framework.

### Ensure LC-MS compatibility

Peptide mixtures must be free of components that interfere with chromatographic separation or ionization.^8^ Ideally, MS compatibility should be considered during experimental planning.

However, when reaction or lysis conditions have been optimized independently of MS compatibility, additional cleanup or buffer-exchange may be required prior to digestion or LC–MS/MS analysis. When buffer conditions are compatible with protease activity, proteins can be digested directly in solution,^4^^,^^7^ which generally provides higher peptide recovery and sequence coverage. In many cases, SDS–PAGE performed during the preliminary assessment step can also serve as a convenient route for sample preparation through band excision followed by in-gel digestion.^44^ Alternative cleanup strategies, including protein-level buffer exchange (e.g., using centrifugal filtration devices) or filter-aided sample preparation (FASP),^45^ may be applied when removal of detergents or chaotropes is required. Protein-level buffer exchange provides a straightforward approach to eliminate incompatible reagents and establish digestion-compatible conditions without introducing additional processing steps. Although FASP is effective for detergent removal, its applicability in PTM-focused analyses should be evaluated with caution, as on-filter processing may affect modification stability or recovery, particularly for labile or low-stoichiometry modifications.

### Disulfide bond reduction and cysteine alkylation

When cysteine residues are present, disulfide reduction and cysteine blocking are commonly incorporated during peptide preparation and introduce defined mass additions that must be considered during database search configuration. In some collaborative workflows, cysteine residues may already be alkylated during upstream sample processing; when this is the case, the corresponding modification mass should be explicitly included in the search parameters. When reduction and alkylation are performed during peptide preparation, disulfide bonds are typically reduced using reagents such as dithiothreitol (DTT) or tris(2-carboxyethyl)phosphine (TCEP), followed by cysteine blocking with alkylating reagents such as iodoacetamide (IAA), as commonly implemented in proteomic sample preparation workflows.^4^^,^^46^ Because these reactions introduce predictable mass shifts on cysteine residues, they are typically treated as fixed or explicitly defined modifications during database search design. However, when unknown modifications are suspected to involve cysteine residues, alkylation strategies should be considered carefully because blocking reactions may mask or alter modification-dependent mass shifts.

### Design a proteolytic strategy

Unknown modifications may block protease cleavage sites, alter peptide solubility, or generate peptides outside optimal detection ranges. Protease selection should therefore be guided by sequence context and the anticipated behavior of the modified region, rather than assuming trypsin is always optimal.^47^ In practice, trypsin often remains a useful starting point because of its robust performance and well-established search frameworks, and semi-tryptic analysis may still recover informative peptides when modification partially disrupts canonical cleavage behavior.

Cleanup procedures should remove incompatible salts, detergents, and residual reagents while minimizing loss of low-abundance modified peptides. After digestion, peptide-level desalting/cleanup (e.g., C18 StageTip) can be used to remove residual small molecules such as urea and salts prior to LC–MS/MS.^48^ If samples are stored after treatment or digestion, modification stability under storage conditions should be verified and documented.

At completion of this stage, peptide mixtures should represent defined comparison conditions, preserve intact or converted modification signatures, and provide sufficient sequence coverage potential for confident site localization. These samples form the substrate for iterative acquisition and data interpretation in Part 3.

## Part 3: LC-MC/MS acquisition and iterative data interpretation

LC–MS/MS acquisition and database interpretation together constitute the analytical core of modification identification when identity or site is unknown. In targeted-protein workflows, LC–MS/MS acquisition and database interpretation should be treated as an integrated and iterative process rather than strictly sequential analytical steps. Acquisition parameters influence search outcomes, and search results guide refinement of acquisition strategy. Confident detection and residue-level localization, therefore, emerge from iterative convergence between spectral evidence, chemical plausibility, and database interpretation.

**Figure 3:**
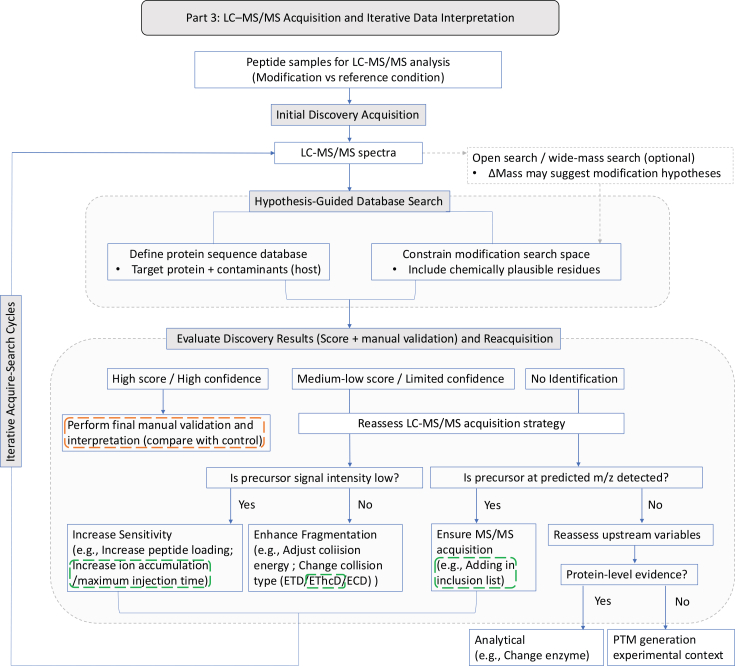
Decision framework for LC–MS/MS acquisition and iterative data interpretation This figure summarizes the iterative acquire–search–refine workflow described in Part 3. Peptide samples from modified and reference conditions are analyzed by discovery LC–MS/MS acquisition and interpreted through hypothesis-guided database searching with controlled protein and modification search spaces. Additional exploratory search strategies may be included where appropriate but are not emphasized in this framework. Search outcomes are evaluated using scoring metrics and manual MS/MS validation. High-confidence identifications proceed to final interpretation, whereas limited-confidence results or absence of identification trigger reassessment of acquisition parameters or upstream experimental variables through iterative acquire–search cycles. The dashed overlays highlight two representative analytical contexts. The green overlay indicates an endogenous or condition-dependent modification scenario, in which analytical challenges are dominated by sensitivity and peptide-level localization. In such cases, iterative refinement of LC–MS/MS acquisition—such as targeted re-acquisition, increased ion accumulation/injection time, improved precursor selection, and optimized fragmentation strategies (e.g., EThcD)—is used to achieve confident identification. In contrast, the orange overlay represents a chemically defined modification scenario, where prior knowledge of reaction chemistry enables constrained search-space design and more direct interpretation, shifting the analytical emphasis toward hypothesis-guided database searching and validation of expected modification patterns. Together with Figure 2, this workflow illustrates how different modification contexts shift the analytical bottleneck across the framework, from experimental design and sample preparation to LC–MS/MS-driven refinement.

This iterative behavior becomes particularly prominent in endogenous or condition-dependent modification scenarios, where sensitivity and localization challenges often necessitate extensive refinement of LC–MS/MS acquisition and interpretation, as illustrated in Figure 3.

### Initial discovery acquisition

#### High-resolution precursor detection

Accurate MS1 measurement is essential for detecting modification-dependent mass shifts, particularly when mass differences are small or stoichiometry is low.^4^^,^^30^ Adequate resolving power improves discrimination of overlapping isotopic envelopes and co-eluting charge states.

All defined comparison conditions must be acquired under identical instrument parameters to preserve interpretability.

#### Fragmentation strategy

Collisional fragmentation (HCD or CID) provides a broadly applicable starting point and typically generates interpretable backbone fragment ion series.^30^

For targeted-protein analyses, data-dependent acquisition (DDA) is generally preferred because it preserves explicit precursor–fragment relationships and supports manual inspection.^49^

The objective of discovery acquisition is not maximal coverage, but detection of candidate modified precursor ions and acquisition of interpretable MS/MS spectra.

### Hypothesis-guided database search strategy for targeted modifications

In targeted-protein workflows, database searching should be treated as a hypothesis-guided and iterative process rather than a purely exploratory step. Constraining the search space—through selection of a defined protein database and controlled formulation of modification models—improves interpretability and facilitates confident identification and residue-level localization.

Open-search or wide-mass-tolerance strategies may serve as optional exploratory tools for detecting candidate mass shifts without predefined modification lists.^16^^,^^17^^,^^50^ However, in practice, meaningful interpretation of open-search results typically requires sufficient signal intensity, recurrence across multiple peptides or sites, and clear condition dependence between experimental and control samples. In studies focused on defined protein targets, limited spectral counts and heterogeneous modification chemistry often further restrict interpretability, and open search alone may yield numerous ambiguous candidates. Accordingly, open search can serve as an initial hypothesis-generating step, but its effectiveness is often limited in targeted workflows. In practice, candidate mass shifts identified by open search typically require further refinement through constrained, chemistry-informed database searching and manual spectral validation to achieve confident modification assignment.^27^

Database searching in targeted-protein workflows involves two conceptually distinct layers: (1) definition of the protein sequence database and (2) construction of the modification search space. Clear separation of these layers improves interpretability and preserves analytical rigor.

#### Protein sequence database design

The protein sequence database defines the competitive search environment. In targeted workflows, searches are often initially restricted to the protein of interest together with a database of common contaminants, which helps maintain realistic score behavior while preserving specificity. Enzyme specificity may be relaxed (e.g., allowing semi-tryptic peptides) to accommodate incomplete digestion or modification-dependent cleavage effects.

If confident identification cannot be achieved under these constrained conditions, the search space can be progressively expanded, for example by including a broader background database such as the host proteome used for recombinant expression. Because searching against an overly narrow database may distort score distributions due to insufficient competition, controlled and iterative expansion of the protein database supports stable statistical evaluation while maintaining interpretability.^15^

#### Modification search space design

Independent of protein sequence selection, the modification search space defines which mass shifts and residue assignments are permitted during scoring. Modification models should therefore be introduced deliberately and iteratively. Candidate modifications should be restricted to chemically plausible residues, the number of simultaneous variable modifications should be limited, and proposed modification models should align with the reaction mechanism and experimental design. In some cases, candidate mass shifts suggested by exploratory analyses such as open or wide-mass searches may also inform the formulation of modification hypotheses. When the modification mass is uncertain but chemical reactivity is partially defined, testing one candidate modification model at a time is often preferable to enabling multiple speculative models simultaneously. Sequential, chemistry-informed refinement improves interpretability and facilitates manual validation. Disciplined control of both the protein sequence database and the modification search space is therefore a defining strength of targeted-protein workflows.

### Evaluation of discovery results

Search outcomes should be interpreted in the combined context of scoring metrics, spectral quality, condition specificity, and chemical plausibility.

Database search results are typically filtered using a false discovery rate (FDR) of ∼1% at the peptide or PSM level to control identification confidence. However, in analyses involving limited numbers of spectra, such thresholds may yield few or no identifications. In these cases, less stringent score-based filtering can be used to retain a broader set of candidate assignments for subsequent manual evaluation (e.g., relaxed ΔCn or analogous score metrics, depending on the search engine).

When site-level interpretation is required, localization confidence may be evaluated using available scoring metrics where applicable; however, in practice, confident site assignment often relies on integration of fragment ion coverage, chromatographic behavior, and experimental context rather than a single localization score. Ambiguous assignments should be explicitly reported rather than excluded.

Database search results generate candidate assignments, but numerical confidence values alone do not ensure correct residue-level localization. In targeted-protein workflows, search configuration and database size can influence scoring behavior; therefore, assignments should be treated as provisional until supported by coherent fragment evidence and chemical logic.

Additionally, evaluation should integrate both database search scores and manual MS/MS inspection, which remains essential for confirming fragment consistency and modification localization.^31^ Following initial evaluation, search results can generally be interpreted in two confidence tiers based on scoring metrics and manual spectral validation.

#### High score/high-confidence identification

If a modified peptide is assigned with strong scoring support and clear fragment coverage, proceed to manual spectrum validation. Confirm fragment continuity across the modified residue and ensure consistency with plausible chemical outcomes. If localization probability remains suboptimal despite strong overall assignment, targeted reacquisition or alternative fragmentation strategies may improve positional confidence. Robust identification requires agreement between scoring metrics, fragment evidence, and chemical plausibility.

#### Medium-low score/limited confidence

If a candidate modified peptide is detected but associated with low scores, limited fragment coverage, or ambiguous localization, first determine the limiting factor.

When precursor intensity is weak, increase sensitivity through greater ion accumulation^49^ or optimization of injected peptide amount within chromatographic and ionization constraints.

When precursor intensity is adequate but fragmentation is insufficient, adjust collision energy, increase accumulation, reduce co-isolation interference, or apply electron-based dissociation methods (e.g., ETD, EThcD, ECD),^51^^,^^52^ particularly for labile or structurally complex modifications.

If localization remains ambiguous despite adequate fragmentation, increase positional coverage using higher MS2 resolution, alternative protease digestion,^47^ or focused reacquisition. In this context, limited statistical support often reflects insufficient fragment evidence rather than the absence of modification.

#### No identification

When independent evidence supports modification presence, absence of peptide-level identification often reflects analytical sensitivity limitations rather than absence of modification. Initial verification should focus on analytical factors, including confirmation of predicted precursor m/z values, charge states, and acquisition range coverage, as well as whether corresponding precursors are selected for MS/MS. If necessary, sensitivity may be improved through increased peptide loading, extended ion accumulation, or targeted inclusion of calculated precursor masses.

When protein-level assessment does not independently support modification, absence of peptide-level detection is less diagnostically specific and may reflect limited modification formation. In such cases, after basic verification of acquisition parameters, reassessment of upstream variables—such as reaction efficiency, modification stability, digestion strategy, or peptide recovery—may be more informative than further adjustment of acquisition settings.

### Iterative acquire-search-refine strategy

Identification of modifications not specified beforehand typically proceeds through iterative refinement.

• Acquire discovery data.
• Perform chemistry-informed constrained search.
• Evaluate spectral quality and localization.
• Refine acquisition parameters or search constraints.
• Re-acquire targeted data.
• Re-search under refined hypotheses.

This iterative cycle continues until spectral evidence and chemical plausibility converge.

### Manual validation and final interpretation

Automated identification scores and localization probabilities provide useful guidance but cannot replace expert spectrum inspection when analyzing modifications whose identity or site is not known beforehand. Manual validation should therefore apply explicit criteria, including continuity of backbone fragment series across the modified residue, expected retention or neutral loss behavior of modification-dependent fragments, absence of conflicting fragment assignments, condition specificity relative to the defined reference state, and consistency with chemically plausible reaction outcomes. High-confidence modification assignment requires convergence of database scores, fragment evidence, and chemical plausibility rather than reliance on any single metric.

To further reduce the risk of overinterpretation, a minimal evidence framework can be applied. A candidate modification should meet the following criteria.

1. **MS/MS spectral support**: Fragment ions support peptide sequence assignment and are consistent with the proposed modification mass shift. Detection of the same modification in related peptide species (e.g., missed-cleavage variants) provides additional supporting evidence for assignment, although such observations are not considered independent validation.
  In some cases, modification-specific fragment features (e.g., characteristic neutral-loss ions) may provide additional supporting evidence, but can also affect database search scores. Such features should therefore be interpreted in conjunction with manual spectral evaluation rather than score alone.
2. **Site localization evidence**: When required, fragment coverage supports residue-level assignment; ambiguous localization should be explicitly noted. Site localization can be supported not only by fragment ion coverage but also, in some cases, by chromatographic separation of positional isomers, where distinct elution profiles correspond to different candidate modification sites (Figure S1).
3. **Control comparison**: The signal is absent or substantially reduced in matched control samples processed in parallel. Absence of fragment ions spanning the modification site, combined with detection of the same signal in control samples, strongly suggests incorrect modification assignment. Representative examples illustrating these scenarios are provided in the supplemental information.
4. **Chemical plausibility**: The observed mass shift and residue specificity are consistent with the expected reaction chemistry. For example, citrullination introduces a mass shift of +0.984 Da, which is isobaric with deamidation; therefore, confident assignment requires consideration of residue specificity (arginine versus asparagine/glutamine), fragment ion evidence supporting site localization, and experimental context, as precursor mass alone is insufficient for discrimination.

When feasible, targeted MS analysis or synthetic peptide standards can provide additional validation.

These criteria are intended to guide analytical confidence in modification assignment and should be interpreted in the context of the specific experimental design and modification mechanism. Representative examples corresponding to these criteria are provided in the supplemental information (Figures S1–S3).

Final interpretation integrates validated spectral assignments with experimental context and available reference knowledge. Observed modification mass, residue specificity, and fragmentation behavior should be compatible with the proposed reaction chemistry and consistent with known protein sequence features or previously reported modification patterns when available. Confidence therefore emerges from agreement among precursor detection, interpretable MS/MS fragmentation, statistically supported database assignments, condition specificity, and chemically plausible modification mechanisms. When these elements converge, LC–MS/MS analysis can support confident residue-level localization of previously uncharacterized protein modifications.

To facilitate practical application of the framework described above, key decision points and evaluation criteria are summarized in Table 1. This table is intended as a concise guide to support analytical decision-making across experimental design, database searching, spectral evaluation, and interpretation, rather than as a prescriptive workflow.

**Table 1:** Practical decision and evaluation guide for targeted analysis of previously uncharacterized protein modifications

Analytical stage	Key question	Recommended approach	Practical guidance
Search strategy	Is modification chemistry partially defined?	Use constrained, hypothesis-guided search	Restrict candidate modifications to chemically plausible residues; evaluate one modification model at a time
–	Is modification mass unknown or unexpected?	Use exploratory (open or wide-mass) search as a hypothesis-generating tool	May suggest candidate mass shifts; generally not well suited for targeted modification analysis, especially for low-stoichiometry or single-site modifications.
–	Are candidate spectra being missed under stringent search constraints?	Maximize candidate retrieval using permissive search settings	Use relaxed score thresholds (e.g., ΔCn) and, where appropriate, restricted search spaces (e.g., single-protein databases) to retain potential matches; prioritize sensitivity at this stage, followed by downstream filtering based on orthogonal criteria.
Database search evaluation	How should candidate identifications be filtered?	Apply context-appropriate filtering	Use FDR where applicable; in low-spectral contexts, relaxed score-based filtering (e.g., ΔCn or analogous metrics) may retain candidates for manual evaluation
–	Is site localization required?	Evaluate localization using integrated evidence	Combine fragment ion coverage, chromatographic separation (LC evidence), and experimental context; report ambiguity when unresolved
MS/MS evaluation	Is the identification supported by spectral evidence?	Perform manual MS/MS inspection	Confirm fragment consistency across the modified region and agreement with the proposed mass shift
–	Does fragmentation support residue assignment?	Assess site-localizing ions	Fragment ions should differentiate candidate modification sites where possible
Control-based interpretation	Is the modification condition-dependent?	Compare matched controls	Signal should be absent or reduced in control samples processed in parallel
–	Could the signal arise from artifacts?	Evaluate control consistency and spectral support	Absence of site-localizing ions together with presence in controls suggests incorrect assignment. When matched controls are insufficient, differential or stepwise labeling strategies may provide additional support for specificity.
Chemical plausibility	Is the assignment chemically reasonable?	Evaluate compatibility with reaction or biological context	Mass shift and residue specificity should align with expected chemistry or biochemical mechanism
Evidence integration	Is the assignment sufficiently supported?	Integrate multiple evidence layers	Combine MS/MS evidence, LC behavior (e.g., separation of positional isomers), control comparison, and consistency across related peptide forms
Failure/no identification	No modified peptide detected?	Reassess detectability	Confirm precursor presence, ionization efficiency, and peptide properties
–	MS1 detected but no MS/MS?	Adjust acquisition strategy	Use inclusion lists, increase ion accumulation, or optimize duty cycle
–	Fragmentation insufficient?	Modify fragmentation conditions	Adjust collision energy or consider alternative fragmentation modes (e.g., ETD/EThcD)

## Discussion: Interpretation, expected outcomes, and limitations

To further illustrate how the framework operates across stages, we consider two representative scenarios that highlight how different modification contexts influence analytical interpretation across the workflow. In a chemically defined covalent modification system (e.g., SuFEx-based chemistry), planning is guided by predictable reaction mechanisms, enabling protein-level assessment and targeted sample preparation strategies, including conversion of bulky adducts into MS-compatible forms. In contrast, in an endogenous or condition-dependent modification scenario (e.g., citrullination-like modifications), protein-level detectability is limited, and analysis proceeds directly to peptide-level processing following protein isolation. In such cases, the primary analytical challenge shifts to LC–MS/MS acquisition and interpretation, where low modification stoichiometry, minimal mass shifts, and lack of direct enrichment make sensitivity and confident localization the dominant analytical constraints, requiring iterative refinement.

Together, these scenarios demonstrate how different modification contexts engage distinct regions of the framework, from front-end experimental design to LC–MS/MS-driven refinement, and consequently influence how modification assignments should be interpreted.

### Contextual interpretation of modification assignments

The workflow described in Parts 1–3 enables confident identification and residue-level localization of protein modifications. Once modification sites have been established by MS-based evidence, interpretation depends on the biological and experimental context of the study. The analytical framework itself does not assume origin or functional significance; rather, it generates molecular evidence that must be contextualized according to the study’s objectives. This section outlines guiding principles for interpreting modification assignments after analytical validation.

Distinguishing true modifications from preparation-induced artifacts relies primarily on control design, condition specificity, and chemical consistency, rather than MS evidence alone.

#### Drug-directed covalent modification studies

In drug-directed covalent studies, the objective is typically to determine whether a compound forms a covalent interaction with a target protein and to identify the modified residue(s).^21^^,^^22^^,^^23^ Interpretation therefore centers on chemical plausibility and condition dependence. Database searches should incorporate reaction outcomes consistent with compound chemistry, including intact adducts, leaving-group loss products, hydrolysis products, or chemistry-dependent conversion products introduced during sample processing.^21^^,^^22^^,^^23^^,^^25^

Matched controls—such as untreated samples, vehicle controls, or inactive analog treatments—are essential to demonstrate treatment dependence.^25^^,^^26^^,^^27^ Reproducible localization of a modification to a specific residue, combined with condition specificity and chemical consistency, supports assignment of a drug-directed covalent interaction.

#### Endogenous or condition-dependent modifications

In studies focused on endogenous or condition-induced modifications, the objective shifts toward identifying PTMs without prior knowledge of chemical identity. Interpretation proceeds iteratively: initial identification of candidate mass shifts may guide refinement of modification hypotheses and targeted database searches.

Absence of prior annotation in curated databases does not preclude biological relevance, as many PTMs remain context-dependent or incompletely characterized.^5^^,^^6^ Across both contexts, several general principles apply. Residue-level MS/MS evidence is required before drawing chemical or biological conclusions,^31^ and interpretation should follow—rather than precede—confident site localization. Comparisons to matched controls distinguish condition-dependent modifications from endogenous PTMs or sample-handling artifacts.

By explicitly separating analytical localization from contextual interpretation, this framework ensures that biological conclusions remain grounded in experimentally supported molecular evidence.

Together, modification assignment in targeted LC–MS/MS workflows is inherently context-dependent and cannot be determined by database search scores alone, but instead emerges from integration of fragment ion evidence, control comparisons, chemical plausibility, and consistency across related observations.

As illustrated by representative cases in the supplemental information, spanning both chemically defined and endogenous modification scenarios, this framework is intended to support high-confidence localization, identify unsupported assignments, and facilitate interpretation of low-abundance or endogenous modifications under realistic experimental conditions.

### Expected analytical outcomes

Application of the framework described in Parts 1–3 enables confident residue-level localization of previously uncharacterized protein modifications using bottom-up LC–MS/MS, even when modification chemistry or site is not predetermined. The workflow prioritizes interpretability and localization confidence over breadth of discovery, allowing investigators to establish condition-dependent modification events on defined protein targets with high evidentiary rigor.

In favorable cases, modified peptides may be detected during initial discovery acquisition. However, low stoichiometry, heterogeneous chemistry, or unfavorable peptide properties frequently require iterative refinement, including targeted re-acquisition, alternative fragmentation strategies, or adjusted digestion approaches. Protein-level mass shifts may be observed when modifications introduce substantial mass changes, but such shifts are neither required nor expected for many biologically relevant PTMs. Absence of gel mobility changes or precursor-level enrichment therefore does not preclude successful peptide-level identification.

When interpreting modification assignments, it is important to recognize that sample preparation strategies may influence both modification stability and detectability. Protein-level buffer exchange, for example, enables removal of incompatible reagents while preserving compatibility with downstream digestion, whereas more extensive processing workflows may introduce additional sources of variability or modification loss.

When successfully applied, the framework supports assignment of specific residues based on high-confidence MS/MS evidence, reinforced by matched controls, chromatographic behavior, and manual spectrum inspection. Importantly, this section addresses analytical performance rather than biological interpretation, which is considered separately.

Collectively, these considerations highlight that analytical success depends on aligning experimental design, detectability, and LC–MS/MS strategy with the underlying modification context, rather than following a single fixed workflow.

### Scope of applicability

This protocol is optimized for proteins with known primary sequences and is not intended for de novo peptide or protein sequencing. The strategy relies on database-driven identification and chemistry-constrained hypothesis testing rather than sequence discovery. Very low-stoichiometry modifications may fall below detection limits without enrichment, targeted acquisition, or increased sampling depth. Highly labile adducts may undergo partial fragmentation during ionization or MS/MS, complicating confident site localization even when precursor ions are detected.

Although primarily designed for identification of previously uncharacterized or non-canonical modifications, the framework also applies to a distinct analytical scenario: cases in which modification chemistry is known, but the modified residue(s) on a defined protein remain undefined. In such studies, the central challenge shifts from defining plausible chemical outcomes to achieving sufficient sensitivity and fragment coverage to localize low-abundance modifications.

Here, analytical emphasis favors depth of sampling rather than proteome-wide breadth. Increased ion accumulation times, refined precursor isolation, targeted inclusion lists, alternative fragmentation strategies, and iterative re-acquisition may be necessary to obtain sufficient evidence for confident localization. Unlike the interpretive scenarios described above, this context primarily reflects analytical optimization rather than biological contextualization.

### Methodological boundaries and validation considerations

Additional limitations arise in complex biological samples, where competing endogenous PTMs, chemical background signals, or sample-handling artifacts may complicate interpretation. These factors underscore the importance of matched controls, careful experimental design, and critical evaluation of LC-MS/MS evidence.

While MS-based analysis provides molecular-level support for modification identity and residue localization, it does not in itself establish chemical mechanism or biological function. Where feasible, complementary approaches—such as targeted MS measurements, comparison to synthetic peptide standards, or orthogonal biochemical assays—may further strengthen confidence in assignment and interpretation.

Overall, characterization of previously uncharacterized modifications remains an iterative, decision-driven process rather than a single analytical step. Integration of experimental design, detectability assessment, LC–MS/MS acquisition, and evidence-based interpretation provides a structured path toward confident residue-level assignment while acknowledging practical limitations in sensitivity, specificity, and modification stability.

## Acknowledgments

The author thanks the editorial team for their valuable guidance, particularly the suggestion to reshape the manuscript into a Primer, which helped improve the clarity and overall organization of this work.

## Author contributions

Conceptualization, methodology, investigation, writing – original draft, and writing – review and editing, Y.Z.

## Declaration of interests

The author declares no competing interests.
