# Supplementary material for: A decision-driven framework for the mass spectrometry analysis of previously uncharacterized protein modifications
Source: STAR Protoc. 2026 May 28;7(2):104567. doi: 10.1016/j.xpro.2026.104567 (PMC13241734; doi:10.1016/j.xpro.2026.104567)
Supplement: Document S1. Figures S1–S3 [file mmc1.pdf]

Supplementary Figures S1–S3 present representative examples of modification assignment, spanning chemically defined and endogenous modification scenarios. Figure S1 illustrates high-confidence site localization supported by fragment ion evidence and chromatographic separation of positional isomers. Figure S2 provides an example of incorrect or unsupported modification assignment, highlighting the absence of site-localizing fragment ions and the presence of comparable signals in control samples. Figure S3 demonstrates context-dependent identification of an endogenous modification, where confident assignment is achieved through integration of fragment evidence, chemical plausibility, and consistency across related peptide forms.

LC–MS/MS analyses were performed using an Ultimate 3000 ultra-high-performance liquid chromatography (UPLC) system (Thermo Fisher Scientific) coupled to an Eclipse Tribrid Orbitrap mass spectrometer (Thermo Fisher Scientific). Raw data were processed using Proteome Discoverer 2.4 (Thermo Fisher Scientific) with Sequest-based database searching. Candidate peptide-spectrum matches (PSMs) were filtered using either a false discovery rate (FDR) of ~1% or relaxed score-based criteria (e.g., ΔCn thresholds), depending on the analytical context. In cases where relaxed filtering was applied to retain candidate identifications, assignments were subsequently evaluated by manual MS/MS inspection and integrated evidence-based criteria.

These examples illustrate application of the minimal evidence framework described in the main text and emphasize that confident modification assignment requires integration of multiple lines of evidence rather than reliance on database search scores alone.

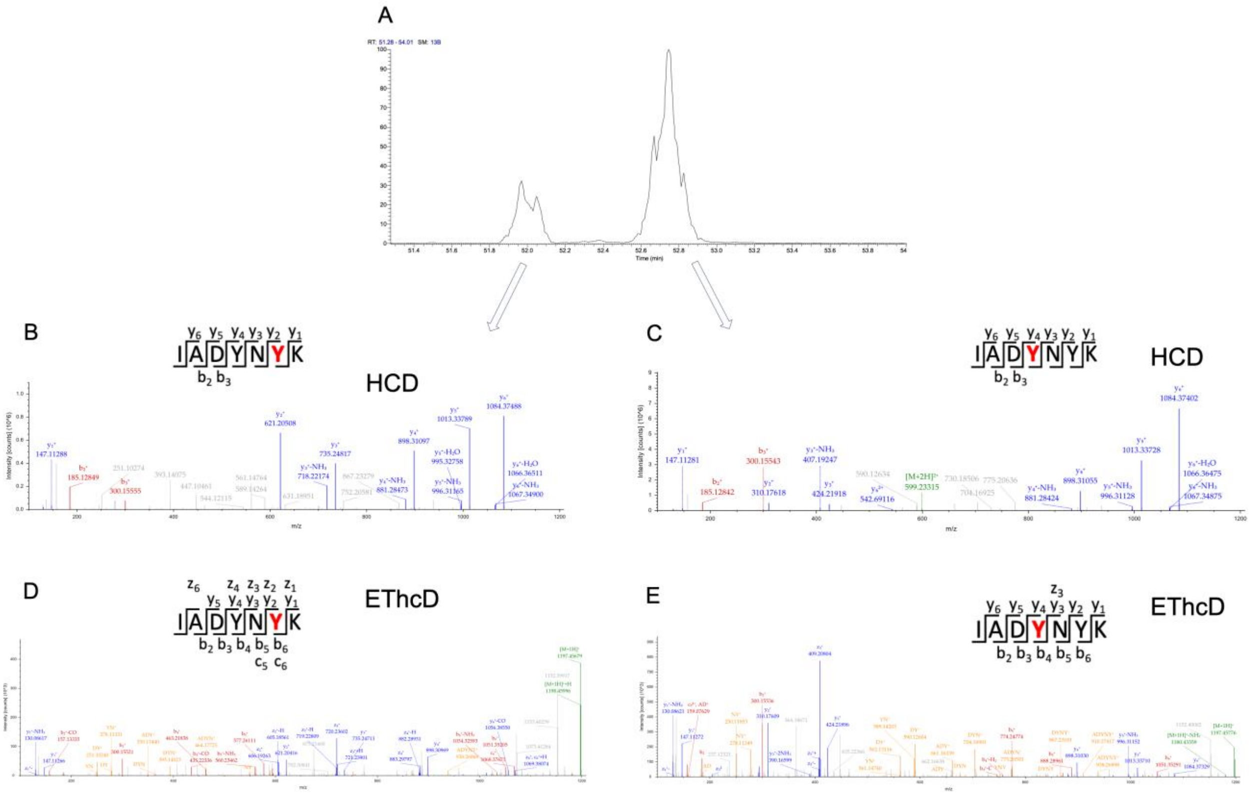

Figure S1. Representative evidence supporting residue-level localization of a modified peptide

Extracted ion chromatograms (A) and annotated MS/MS spectra acquired under HCD (B/C) and EThcD conditions (D/E) are shown for a representative modified peptide.

In this example, b- and y-type fragment ions obtained under HCD provide sufficient coverage to support residue-level localization of the modification. EThcD fragmentation yields additional fragment ions and increased sequence coverage, which may further strengthen confidence in site assignment.

Notably, the extracted ion chromatogram reveals two partially resolved peaks corresponding to distinct positional isomers, each associated with a different localization pattern. This chromatographic separation provides independent supporting evidence for site assignment in addition to MS/MS fragmentation.

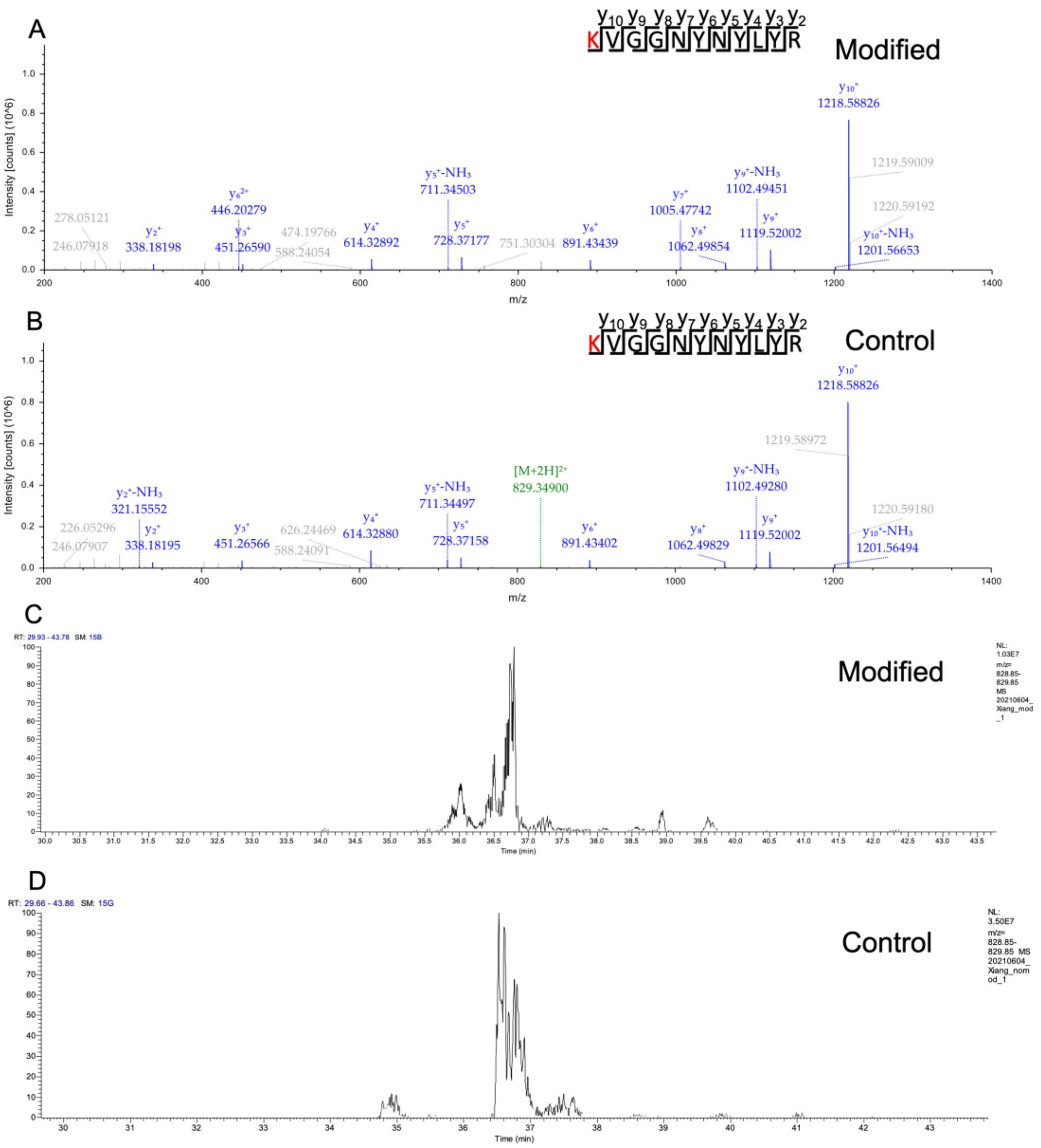

Figure S2. Example of ambiguous modification assignment lacking site-localizing evidence.

Annotated HCD MS/MS spectra (A/B) and extracted ion chromatograms (C/D) are shown for a candidate modified peptide detected under both modified and control conditions.

In this example, fragment ion coverage is limited to y-type ions and does not include ions spanning the proposed modification site, precluding confident residue-level localization. In addition, a comparable signal is observed in the matched control sample, indicating a lack of condition dependence.

Together, these features suggest that the proposed modification assignment is not supported by sufficient analytical evidence and likely represents an ambiguous or false-positive identification.

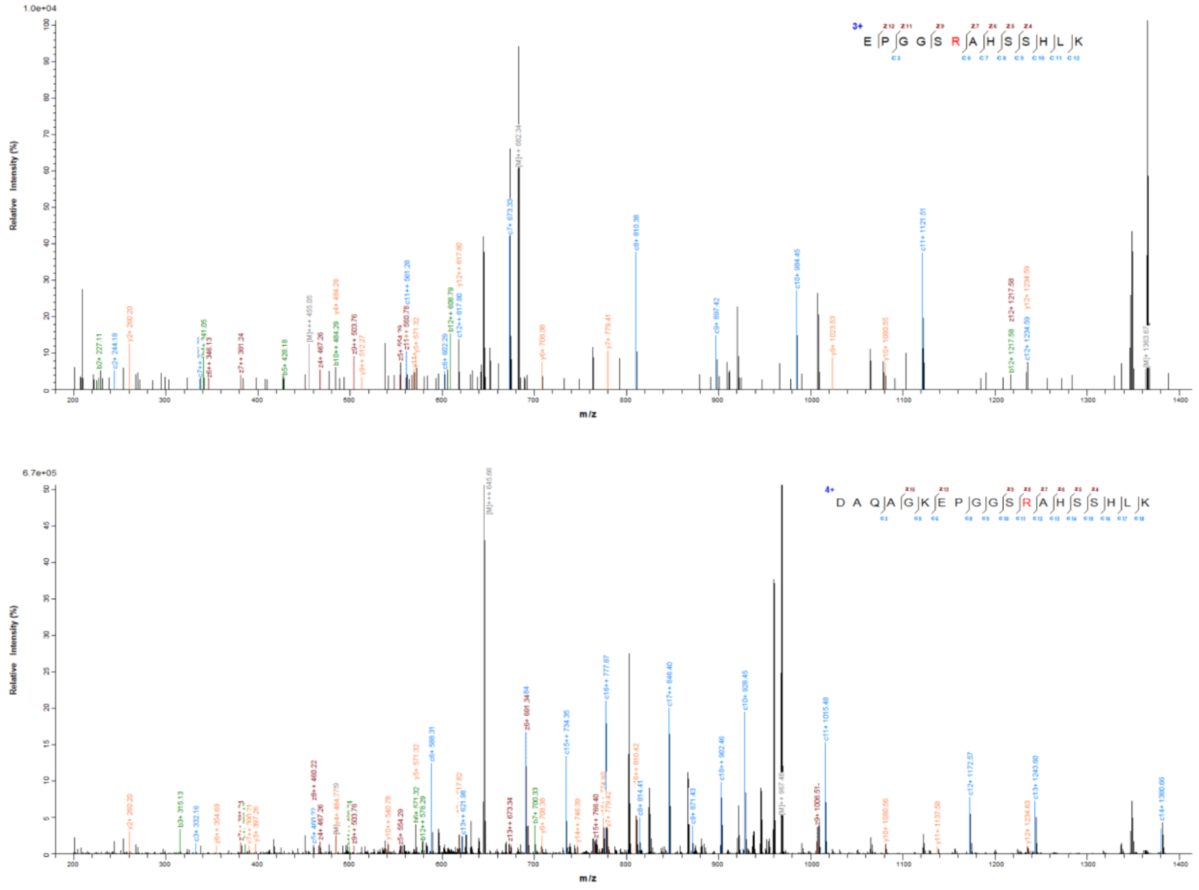

Figure S3. MS/MS evidence supporting site localization of a citrullinated peptide across related peptide forms

Annotated MS/MS spectra of a citrullinated peptide detected as a fully tryptic peptide (top) and a missed-cleavage variant (bottom) are shown. Fragment ions in both spectra are consistent with the peptide sequences and support assignment of the observed +0.984 Da mass shift to the proposed arginine residue.

Citrullination introduces a mass shift that is isobaric with deamidation; therefore, confident assignment requires integration of residue specificity, fragment ion evidence supporting site localization, and experimental context.

Detection of the same modification in both fully tryptic and missed-cleavage peptide forms provides consistent supporting evidence for modification assignment, indicating that the observed mass shift is reproducible across related peptide species. Although such observations are not considered independent validation, they strengthen confidence in modification assignment when combined with fragmentation evidence.
